# Validity and reliability of the Turkish version of the parent perceptions of physical activity scale

**DOI:** 10.55730/1300-0144.5646

**Published:** 2023-03-27

**Authors:** Kamile UZUN AKKAYA, Müjde ÇALIKUŞU İNCEKAR, Bülent ELBASAN

**Affiliations:** 1 Department of Physiotherapy and Rehabilitation, Faculty of Health Sciences, Gazi University, Ankara, Turkey; 2 Department of Pediatric Nursing, Faculty of Health Sciences, Yüksek İhtisas University, Ankara, Turkey

**Keywords:** Neurodevelopmental disorders, physical activity, parents, reliability, validity

## Abstract

**Background/aim:**

The Parent Perceptions of Physical Activity Scale (PPPAS) is a scale developed to measure the physical activity perceptions of parents of children with neurodevelopmental disorders about their children. Turkish version of the PPPAS has yet to be established. The purpose of the present study is to examine the validity and reliability of the Turkish version of the PPPAS.

**Material and methods:**

The study included 130 parents with neurodevelopmental children. In the validity analyses of PPPAS, language validity, content validity analysis, and confirmatory and explanatory factor analysis were performed for construct validity. In the reliability analyses, Cronbach alpha analysis was used for internal consistency analysis, and intraclass correlation (ICC) analysis was used for test retest.

**Results:**

The validity index was calculated as 0.94. Since the factor loading of the three questions in the survey remained below 40%, these questions were removed. Construct validity was achieved for two primary subscales of the PPPAS. It was found that the ICC values for the reliability analysis of the PPPAS showed a perfect fit at the level of 0.918 for the benefits of the physical activity subdimension, and the physical activity barriers subdimension showed a perfect fit at the level of 0.916 (p = 0.001).

**Conclusion:**

The Turkish PPPAS, which consists of two subscales, namely the benefits and barriers of physical activity, is valid and reliable. This tool can measure the physical activity perceptions of parents with preschool-age children with neurodevelopmental disorders in the Turkish population.

## 1. Introduction

Neurodevelopmental disorder is an impairment of the brain and central nervous system. There are deficiencies in some skills, such as motor development problems, sensory integration disorders, language and speech retardation, learning difficulties, weakness in organized skills, behavioral problems, and communication problems in children with neurodevelopmental disorders [1]. Physical activity is fundamental to general health and motor development from birth and has many benefits for children in terms of physical, psychosocial, cognitive, and emotional aspects [2]. In addition, physical activity reduces the risk of chronic diseases such as obesity and diabetes, which are often encountered today [3]. Physical activity levels in children are influenced by many personal, environmental, and familial factors. There is a difference in physical activity levels between children with disabilities and those with typical development. The physical activity levels of children with neurodevelopmental disorders and impaired motor development are negatively affected. For this reason, children are at greater risk of secondary health problems such as obesity [4,5]. Parents’ attitudes and behaviors regarding physical activity, both in children with typical development and in children with disabilities, have an essential role in the development of positive health behaviors in children [6]. It is known that the physical activity beliefs of families and the physical activity behaviors of children are directly related [7].

Children with neurodevelopmental disorders depend more on their families to be active than their healthy peers. It has been reported that the parents of a child with a neurodevelopmental disorder have a more significant impact on their child’s physical activity levels than those of children with typical development [8]. It is known that parents who believe in the benefits of physical activity are role models for their children in terms of physical activity. These parents encourage their children to do physical activity and have disabled children who are more active in life [6]. Parents of children with neurodevelopmental disorders may exhibit very protective behaviors towards their children from time to time [9,10]. In addition, parents’ concerns about their children’s safety, competence, and exclusion from society may prevent them from increasing their children’s physical activity levels [6]. Therefore, understanding physical activity in children with disabilities from the parents’ perspective and investigating the factors that parents see as facilitators and barriers to physical activity can help develop relevant interventions to increase the activity levels of children with disabilities.

Parent Perceptions of Physical Activity Scale (PPPAS) was developed for children with neurodevelopmental disorders to measure parents’ perceptions of physical activity of their children [11]. There needs to be a Turkish validity and reliability scale in the literature on this subject. Thus, this study aims to determine the validity and reliability of PPPAS in Turkish.

## 2. Methods

### 2.1. Study design

**Figure 1:**
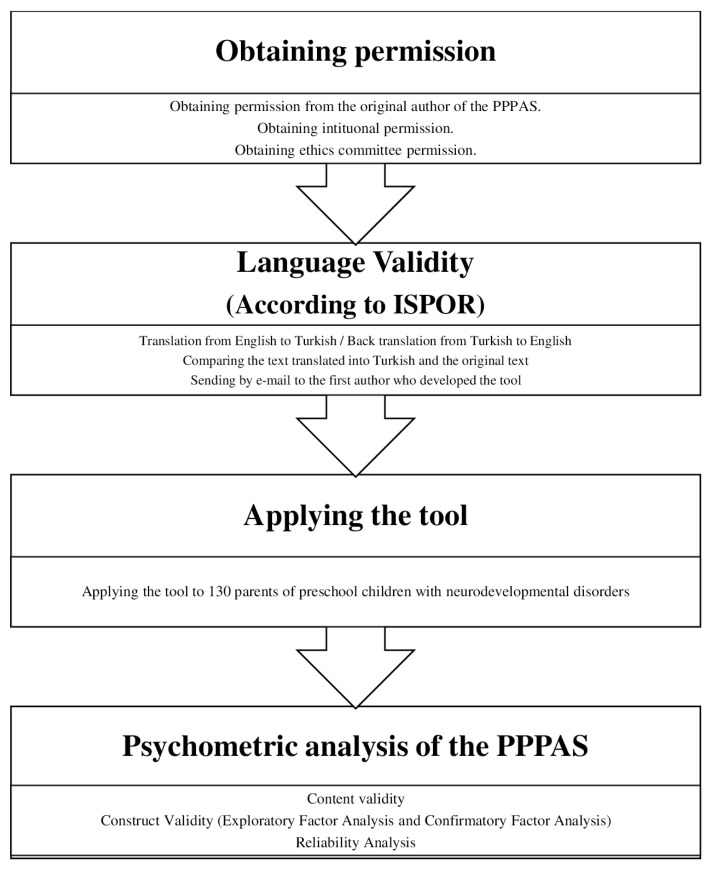
The flowchart of the study.

The study was carried out at Gazi University, Faculty of Health Sciences, Department of Physiotherapy and Rehabilitation, Pediatric Rehabilitation Unit, between September 2021 and June 2022. Figure 1 shows a flowchart summarizing the work order (Figure 1). The ethics committee permission required for the study was obtained from Yüksek İhtisas University, Non-Interventional Research Ethics Committee (date: 09.10.2020, no: 2020/11/01). A written consent form was obtained from the parents who agreed to participate in the study. After obtaining permission from the scale’s authors, PPPAS was translated into Turkish and culturally adapted by international rules [12].

### 2.2. Participants

Parents of preschool children with neurodevelopmental disorders aged 2–6 years were included in the study. Parents who did not speak Turkish and could not read and write were excluded from the study.

The sample calculation was made with the G*Power 3.1.9.2 package program [13]. While determining the sample size in scale studies, the rule that it should be at least five times the number of items in the scale was considered. Considering the possible missingness and measurement errors, the validity and reliability study of the 25-item PPPAS was conducted with a total of 130 children [14]. Assuming a test-retest correlation of 0.50 (ρ) and taking Power: 0.80 and α: 0.05, the sample size for the test-retest was 29 [15]. Considering the possible missingness, 30 people were included in the study.

### 2.3. Data collection

Parents were asked to fill out the PPPAS and a form containing demographic information such as their children’s age, height, weight, gender, and illness, in addition to their information such as age and educational status. It took parents approximately 15 min to complete the data collection forms.

#### 2.3.1. Parent perceptions of physical activity scale

The PPPAS was developed by Lakes et al. to measure parents’ perceptions of physical activity of their children. Two scale versions can be applied to infants and preschool children [11, 16]. The PPPAS-preschool version was used in this study. In the first stage, PPPAS-Preschool consisted of 40 pilot items, but based on the analysis result, the questionnaire was reduced to 25 items. The items in the scale consist of two subscales to assess the perceived benefits and barriers to physical activity (18 for the Benefits scale and 7 for the Barriers scale). Items 1, 3, 5, 7, 8, 10, 11, 12, 13, 14, 17, 18, 19, 20, 21, 22, 23, and 24 constituted the benefits of physical activity, and 2, 4, 6, 9, 15, 16, 25 items constituted the barriers of physical activity subscale. The internal consistency coefficients were good or excellent for the subscales of the final 25-item PPPAS. The Cronbach’s alpha value of the barriers subscale of the original scale was 0.83, and the Cronbach’s alpha value of the benefits subscale was 0.95. The 4-point Likert scale is used in the scale, and the items are scored as “Strongly disagree = 1, Disagree = 2, Agree = 3, Strongly agree = 4”. [11].

### 2.4. Language validity (translation process)

The scale’s translation and cultural adaptation procedure were carried out per the Guidelines of the International Society for Pharma economics and Outcome Research (ISPOR) [12]. First, the original tool was translated into Turkish separately by two independent native speakers/translators. It was then evaluated by the researchers and made into a single form. The questionnaire was back-translated from Turkish to English by a person who speaks both languages at a native level. The researchers compared the text translated into Turkish and the original text. Then it was sent by e-mail to the first author who developed the tool, and her approval was received. After the necessary corrections, the language validity of the questionnaire was ensured.

### 2.5. Data analysis

The NCSS (Number Cruncher Statistical System) 2007 (Kaysville, Utah, USA) and Lisrel 8.8 program were used for statistical analyses. While evaluating the study data, descriptive statistical methods (mean, standard deviation, median, first quarter, third quarter, frequency, percentage, minimum, maximum) were used. The Student t-test was used in paired group comparisons, while Oneway Anova test was used in comparisons of three or more groups. For the validity analyses of the scale, content validity index, explanatory factor analysis (EFA), and confirmatory factor analysis (CFA) were performed. For the reliability analysis of the scale, Intraclass Correlation Coefficient (ICC) and Cronbach’s alpha analyses were performed.

#### 2.5.1. Content validity

Five experts evaluated the Turkish version of the questionnaire regarding scope validity. The experts evaluated the questionnaire items according to the Content Validity Index (CVI). This index is of the Likert type and includes the response of each item as “Not relevant = 1” and “Highly relevant = 4”. The calculation was made when experts gave 3 or 4 points to each item. [17]. The reliability among independent evaluators was accepted as 0.90 “excellent”, 0.80 “very good”, and 0.70 “adequate” [18]

#### 2.5.2. Construct validity

EFA and CFA were applied for the scale’s construct validity.

#### 2.5.3. Reliability analysis

Reliability analysis was evaluated by test-retest analysis. The scale was readministered to 30 parents who agreed to complete the questionnaire a second time one week after the first application, and reliability analysis was performed with ICC and Cronbach’s alpha analyses.

## 3. Results

This study was conducted with the parents of 130 children, 64.6% (n = 84) were boys, and 35.1% (n = 46) were girls. The mean age of the children was determined as 4.35 ± 1.42 years. Of the parents who completed the questionnaire, 63.8% (n = 83) were mothers, and 36.2% (n = 47) were fathers. Demographic information of the parent and child is given in Table 1.

### 3.1. Content validity of the parent perceptions of physical activity scale

In this study, the content validity was calculated as 0.94.

### 3.2. Construct validity of the parent perceptions of physical activity scale

#### 3.2.1. Exploratory factor analysis

EFA was used for the construct validity of PPPAS. When Quartimax rotation was applied in EFA, it was observed that the questions were collected under two factors and the explanatory coefficient was 48.54%. As a result of the factor analysis, two questions with factor loadings below 40% were determined (2nd and 3rd question), and it was decided to remove them from the scale.

When Quartimax rotation was applied to the PPPAS again in the EFA analysis with the remaining 23 questions as the second stage, it was observed that the questions were gathered under two factors and the explanatory coefficient was 51.5%. Due to factor analysis, another question was determined with a less than 40% factor loading (4th question) and decided to remove from the scale.

It was observed that when Quartimax rotation was applied to PPPAS again in EFA with the remaining 22 questions as the third stage, the questions were again collected under two factors and the explanatory coefficient was 53.3%. As a result of the factor analysis, no questions were found that had loads on factors below 40% and loads close to multiple factors (below 10%). In addition, it was concluded that there were no questions with antiimage correlations below 0.500 and that the final version of the scale was in this way.

The Kaiser Mayer Olkin (KMO) sample adequacy measurement value was determined to be 0.900. The Bartlett Sphericity test result, which showed that the data were suitable for EFA, was statistically significant (χ2 = 1800, 407; df = 231, p = 0.001).

When the Equamax rotation was applied in the EFA of the subdimension, it was concluded that the questions were collected under two factors (benefits and barriers to physical activity). The first factor alone explained 43.7% of the total variance, and the first and second factors explained 53.3% of the total variance. Questions 1, 5, 7, 8, 10, 11, 12, 13, 14, 17, 18, 19, 20, 21, 22, 23, and 24 constituted the benefits of physical activity, and 6, 9, 16, 15, 25 questions constituted the barriers of physical activity subfactors in the original PPPAS. After removing the questions in the Turkish version of the PPPAS, items 1, 2, 4, 5, 7, 8, 9, 10, 11, 14, 15, 16, 17, 18, 19, 20, 21 formed the benefits of physical activity and items 3, 6, 12, 13, 22 formed the barriers of physical activity subscale (Appendix).

#### 3.2.2. Confirmatory factor analysis

**Figure 2:**
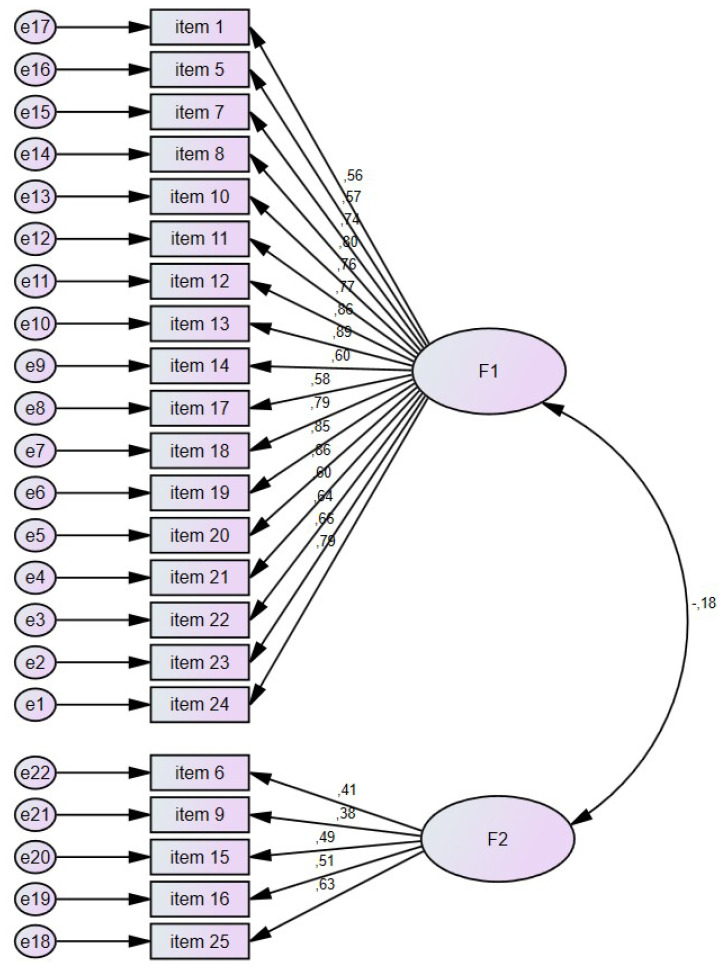
Confirmatory factor analysis graph of Parent Perceptions of Physical Activity Scale.

The standardized loads of the 22 questions related to PPPAS and the questions constituting the two subdimensions in the CFA result are given in Figure 2. The fit criteria (Goodness-of-Fit Indices and corrected Chi-square (ℵ2/df value) for the dimensions in the model established to test the CFA are given in Table 2. The corrected chi-square value showed a good fit [19]. Also, the RMSEA, NNFI, CFI, IFI, and SRMR criteria from other fit criteria showed acceptable fit [20]. Accordingly, it was concluded that our data had a good fit, and our model was statistically significant and valid since the fit criteria showed a good and acceptable fit (p = 0.001) (Table 2).

### 3.3. Reliability of the parent perceptions of physical activity scale

When the Cronbach’s alpha values of the PPPAS were examined, Cronbach’s alpha value was determined as 0.950 for the benefits of physical activity subdimension, 0.603 for the barriers of physical activity subdimension, and 0.899 for the total value.

It was found that the ICC values of the PPPAS showed excellent fit at the level of 0.918 for the benefits subdimension of physical activity and excellent fit at the level of 0.916 for the barriers subdimension of physical activity (p = 0.001) (Table 3).

### 3.4. Identifying results of the parent perceptions of physical activity scale

The average score for the benefits of physical activity was 2.02 *±* 0.52, while the average score for the barriers of physical activity was 3.41 *±* 0.47. The total score on the scale ranged between 1.45 and 3.82, and the average score was found to be 3.09 *±* 0.36 (Table 4).

There was no difference between the scores of the physical activity scale subdimensions according to the people who answered the questionnaire and the presence of siblings (p > 0.05). There was a significant difference between the questionnaire’s physical activity benefit scores according to maternal and paternal education levels (p < 0.05). When the significance was examined, the scores of university graduates were significantly higher (p < 0.05). It was concluded that the physical activity barrier scores did not differ according to the educational status of both the mother and the father (p > 0.05) (Table 5).

## 4. Discussion

In our study, in which we aimed to examine the Turkish validity and reliability of the PPPAS developed by Lakes et al. [11] in preschool children with neurodevelopmental disorders, it was concluded that the Turkish version of the PPPAS was valid and reliable. There needs to be a validity and reliability analysis of the questionnaire in other languages in the literature. ISPOR guide was used for the language validity of the questionnaire, and the questionnaire was translated into Turkish following the Turkish language rules and in a way that is understandable [12]. Five experts conducted the scope validity of the survey. Kline et al. [18] evaluated values over 0.90 as excellent for content validity. This study calculated the CVI value as 0.94, and the scope validity was achieved.

After the language and content validity of the questionnaire, the construct validity was tested. The researchers removed the questions in the original questionnaire whose factor loading was below 40 [11, 21]. Since the factor loading of three questions remained below 40%, the questions were removed and the questionnaire was evaluated over 22 questions [11, 21]. The Barlett Sphericity test result, which showed that the data were suitable for EFA, was also found to be statistically significant (p < 0.01). As a result of the Equamax rotation analysis, it was determined that the questionnaire items were collected under two subfactors, namely the benefits and barriers of physical activity, as in the original version of the questionnaire. The benefits of the physical activity subfactor alone explained 43.7% of the total variance, and the benefits and barriers of physical activity explained 53.3%. In the original version of the questionnaire, it was reported that the benefits of physical activity explained 42.76% of the variance, the barriers to physical activity explained 12.55%, and the total variance explained 55.30% for the 25-item scale [11].

For the reliability analysis of the questionnaire, the same participants were subjected to a test-retest at intervals of 1 week. Koo et al. reported that the ICC value above 0.90 is a perfect fit [22]. It was determined that ICC values for the benefits subdimension of physical activity of PPPAS showed perfect fit at the level of 0.918, and for the barriers subdimension of physical activity, it showed perfect fit at the level of 0.916. In addition, Cronbach’s alpha values of the questionnaire were found to be 0.950 for the benefits sub-dimension of physical activity, 0.603 for the barriers subdimension of physical activity, and 0.899 for the total value. In the original questionnaire, the benefits of physical activity subdimension were determined as 0.95, and the barriers subdimension was determined as 0.83 [11]. The literature has reported that a Cronbach’s alpha value between 0.70 and 0.95 is acceptable [23, 24]. In the study, both the ICC values of the questionnaire show perfect fit, and the total Cronbach alpha value is above 0.70, which indicates that the Turkish version of the PPPAS is reliable.

In this study, mothers and fathers gave similar answers to the questions of the PPPAS questionnaire. Pitchford et al. [7] reported in their study that parental support and encouragement for boys and girls with disabilities may differ; however, there are not many studies in the literature comparing the physical activity perceptions of parents on behalf of their disabled children. Future studies can be carried out on this issue. In the literature, it has been reported that the presence of siblings makes the siblings of disabled children role models and that their siblings encourage the children to participate in physical activity [25,26]. However, our study concluded that parents with disabled children have other children who do not change their perception of the benefits and barriers of physical activity. Due to the absence of a question about the siblings’ presence in the questionnaire, parents’ results may have been similar. In the study, maternal and paternal educational status affected the questionnaire answers. Mothers and fathers with a university degree about physical activity gave more positive answers in the benefits of physical activity section. According to the findings from surveys such as the National Health Interview Survey, individuals with a lower level of education have a lower prevalence of physical activity [27, 28]. A study reported that individuals with sufficient knowledge about physical activity have more positive thoughts about physical activity [29]. It should not be forgotten that parents’ perceptions about physical activity affect their children’s participation in physical activity. In the future, parents of children with neurodevelopmental disorders, especially those with low education, should be provided with informative training on the benefits of physical activity.

In the literature, it has been stated that the physical activity levels of children with neurodevelopmental disorders are lower compared to children with typical development. The participation of families in physical activity and their beliefs about physical activity significantly impact their children [6,8]. For this reason, it is very valuable to measure families’ perceptions of physical activity and to make appropriate interventions. To date, there has been no Turkish questionnaire measuring the physical activity perceptions of families of children with neurodevelopmental disorders. The Turkish validity and reliability of this questionnaire will enable the determination of physical activity perceptions of families with children with neurodevelopmental disorders and will help them to follow an appropriate path, thus contributing to the literature. The Turkish validity and reliability of this questionnaire will be helpful for families with children with neurodevelopmental disorders to determine their physical activity perceptions and to follow an appropriate path. Thus, it will contribute to the literature.

### 4.1. Limitations

Most of the children included in the study were diagnosed with cerebral palsy, and the small number of children with other neurodevelopmental disorders was a limitation of the study. In addition, most of the parents who filled out the questionnaire were mothers. This study may have assessed more mothers’ perceptions of physical activity.

## 5. Conclusion

The Turkish PPPAS, consisting of two subscales, namely the benefits and barriers of physical activity, evaluated on 22 items, is valid and reliable. This tool can measure the physical activity perceptions of parents with children with neurodevelopmental disorders aged 2–6 years in the Turkish population.

## Figures and Tables

**Table 1:** Distribution of descriptive characteristics of the parents and children.

		n (%)
Parents		
Sibling presence	Yes	86 (66.2)
	No	44 (33.8)
Mother education	Primary	34 (26.2)
	High school	38 (29.2)
	University	50 (38.5)
	Graduate	8 (6.2)
Father education	Primary	24 (18.5)
	High school	53 (40.8)
	University	49 (37.7)
	Graduate	4 (3.1)
Age	Mean *±* SD	29.22 ± 6.14
	Median (Min-max)	29 (18–52)
Children		
Sex	Female	46 (35.4)
	Male	84 (64.6)
Child’s disease (%)	Cerebral palsy	102 (78.5)
	ASD	4 (3.1)
	Down syndrome	3 (2.3)
	Rett syndrome	2 (1.5)
	Other diseases	19 (14.6)
Age (years)	Mean *±* SD	4.35 ± 1.42
	Median (Min-max)	4 (2–6)
Height (cm)	Mean *±* SD	103.82 ± 13.88
	Median (Min-max)	104 (75–141)
Weight (kg)	Mean *±* SD	16.89 ± 5.58
	Median (Min-max)	18 (8–41)

Cm: Centimeter; kg: Kilogram; SD: Standard deviation; Min: Minimum; Max: Maximum; ASD: Autism spectrum disorder

**Table 2:** Fit criteria of Parent Perceptions of Physical Activity Scale confirmatory factor analysis.

Fit Indices	Good fit reference values	Acceptable fit reference values	Model results
RMSEA	0 < RMSEA < 0.05	0.05 ≤ RMSEA ≤ 0.10	0.054
NFI	0.95 ≤ NFI ≤ 1	0.90 ≤ NFI ≤ 0.95	0.86
NNFI	0.97 ≤ NNF ≤ 1	0.95 ≤ NNFI ≤ 0.97	0.95
CFI	0.97 ≤ CFI ≤ 1	0.95 ≤ CFI ≤ 0.97	0.96
IFI	0.97 ≤ IFI ≤ 1	0.95 ≤ IFI ≤ 0.97	0.96
RFI	090 ≤ RFI ≤ 1	0.85 ≤ RFI ≤ 0.90	0.83
SRMR	0 ≤ SRMR ≤ 0.05	0.05 ≤ SRMR ≤ 0.10	0.063
GFI	0.95 ≤ GFI ≤ 1	0.90 ≤ GFI ≤ 0.95	0.86
AGFI	0.90 ≤ AGFI ≤ 1	0.85 ≤ AGF I ≤ 0.90	0.81
ℵ^2^/df (265.118/192)	0 ≤ ℵ^2^/df ≤ 2	2 ≤ ℵ^2^/df ≤ 3	1.381

χ2: Chi-Square fit test; df: Degree of freedom; RMSEA: Root mean square error of approximation; NFI: Normed fit index; NNFI: Non-normed fit index; CFI: Comparative fit index; IFI: Incremental fit index; RFI: Revel’s functional index; SRMR: Root mean square residual; GFI: Goodness-of-fit index; AGFI: Adjusted goodness-of-fit index

**Table 3:** Parent Perceptions of Physical Activity Scale test-retest results.

	Test-retest (ICC)	95% CI	p
Benefits of physical activity	0.918	0.835–0.960	**0.001**
Barriers to physical activity	0.916	0.832–0.959	**0.001**

ICC: Intraclass correlation coefficient; CI: Confidence interval

**Table 4:** Evaluation of subdimensions and total scores of Parent Perceptions of Physical Activity Scale.

Subscale	Median (Min-max)	Mean *±* SD
Benefits of physical activity	3.41 (1.24–4.00)	3.41 *±* 0.47
Barriers to physical activity	2.00 (1.00–3.60)	2.02 *±* 0.52
Total	3.09 (1.45–3.82)	3.09 *±* 0.36

Min: Minimum; Max: Maximum; SD: Standard deviation

**Table 5:** Comparison of descriptive characteristics of parents according to Parent Perceptions of Physical Activity Scale scores.

		Physical Activity Benefits (Mean *±* SD)	p	Physical Activity Barriers (Mean *±* SD)	p
Who filled out the questionnaire	Mother	3.39 ± 0.43	a0.666	2.05 **±** 0.56	a0.426
	Father	3.43 ± 0.52		1.97 **±** 0.44	
Sibling presence	Yes	3.42 ± 0.44	a0.555	1.99 **±** 0.53	a0.394
	No	3.37 ± 0.52		2.07 **±** 0.49	
Mother education	Primary	3.35 ± 0.47	b **0.028** *	2.13 ± 0.54	b0.349
	High school	3.28 ± 0.41		1.97 ± 0.46	
	Graduate	3.52 ± 0.47		1.98 ± 0.54	
Father education	Primary	3.17 ± 0.45	b **0.022** *	2.04 ± 0.52	b0.540
	High school	3.43 ± 0.42		2.07 ± 0.47	
	Graduate	3.48 ± 0.49		1.96 ± 0.57	

SD: Standard deviation

a Student t-test,

b Oneway Anova test

* p < 0.05

## Appendix

The Turkish version of the Parent Perceptions of Physical Activity Scale.

**Table:**

	Kesinlikle Katılmıyorum	Katılmıyorum	Katılıyorum	Kesinlikle Katılıyorum
1. Egzersiz alışkanlıklarım, **çocuğumun** hayatı boyunca geliştireceği egzersiz alışkanlıklarını güçlü bir **şekilde** etkileyecektir.				
2. Aktivite **çocuğumun** kalp-damar sisteminin işleyişini geliştirir.				
3. **Çocuğumun** spor veya fiziksel aktivitelere katılma becerisi konusunda endişeleniyorum.				
4. Fiziksel aktivite **çocuğumun** zihinsel uyanıklığını arttırır.				
5. Aktivitenin artması **çocuğumun** fiziksel formda olma seviyesini artırır.				
6. Fiziksel aktivite **çocuğumu** sinirlendirecektir.				
7. Fiziksel aktivite **çocuğumun** kas gücünü arttırır.				
8. Egzersiz yapmak çocuğumun geceleri daha iyi uyumasına yardımcı olur.				
9. **Çocuğumun** fiziksel dayanıklılığı, onu aktif olmaya teşvik ederek gelişir.				
10. Fiziksel aktivite çocuğumun esnekliğini geliştirir.				
11. Egzersiz konusundaki tutumlarım, çocuğumun hayatı boyunca egzersize karşı tutumunu güçlü bir şekilde etkileyecektir.				
12. **Çocuğum** grup halinde yapılan fiziksel aktiviteye veya spor programlarına katılamıyor.				
13. Fiziksel aktivitenin, **çocuğumun** hayal kırıklığına uğramasına yol açacağından korkuyorum.				
14. **Çocuğum** fiziksel aktivite sayesinde kendini daha iyi hissediyor.				
15. Fiziksel aktivite **çocuğuma** kişisel başarı hissi verir.				
16. **Çocuğumu** fiziksel aktivite konusunda cesaretlendirerek gelecekteki sağlığını geliştireceğim.				
17. **Çocuklukta** yapılan fiziksel aktivite **çocuğumu** daha sağlıklı kılacaktır.				
18. **Çocuğumu** aktif bir **çocuk** olmaya teşvik edersem daha uzun yaşayacaktır.				
19. Fiziksel aktivite **çocuğum** için iyi bir eğlencedir.				
20. Egzersizlere verdiğim değer, **çocuğumun** aktif olmasını etkileyecektir.				
21. Fiziksel aktivite **çocuğumun** tüm vücut işleyişini geliştirir.				
22. **Çocuğum** grup sporu ya da aktivite programına katılırsa, diğer **çocuklar** tarafından kabul edilmeyeceğinden endişeleniyorum.				

The PPPAS-Preschool. Note: Items 1, 2, 4, 5, 7, 8, 9, 10, 11, 14, 15, 16, 17, 18, 19, 20, 21 for the Benefits subscale and items 3, 6, 12, 13, 22 for the Barriers subscale.
